# Interactions between N, P in the overlying water and flooding-induced decomposition of *Cynodon dactylon* in the water-level fluctuation zone

**DOI:** 10.3389/fpls.2025.1526507

**Published:** 2025-02-10

**Authors:** Jitao Huang, Ze Luo, Zuopeng Xu, Yanxue Jiang, Jinsong Guo

**Affiliations:** ^1^ Key Laboratory of the Three Gorges Reservoir Region’s Eco-Environment, Ministry of Education, Chongqing University, Chongqing, China; ^2^ College of Environment and Ecology, Chongqing University, Chongqing, China

**Keywords:** water-level fluctuation zone (WLFZ), flooding, plant decomposition, nitrogen and phosphorus, water quality

## Abstract

During flooding in the Water Level Fluctuation Zone (WLFZ), nutrient levels of nitrogen (N) and phosphorus (P) in the overlying water fluctuate due to soil nutrient release, impacting the decomposition of plants like *Cynodon dactylon*. However, limited research on the effects of these nutrient changes on plant nutrient release and water dynamics complicates accurate assessments of water quality impacts. This study used 8 water samples with varying initial nutrient levels to simulate N and P changes induced by WLFZ soil nutrients and examined the decomposition and nutrient dynamics of *Cynodon dactylon*. Results showed that flooding significantly increased initial levels of N and P, especially as particulate nitrogen (PN) and particulate phosphorus (PP), affecting both plant decomposition and nutrient dynamics in the water. After 60 days, *Cynodon dactylon* lost 47.97%-56.01% dry matter, 43.58%-54.48% total nitrogen (TN), and 14.28%-20.50% total phosphorus (TP). Initial PN and total dissolved nitrogen (TDN) promoted dry matter loss, PN and PP promoted TP loss, while PN and TDN inhibited TN loss. By day 60, no positive correlation was found between plant-released N and P and TN or TP in the overlying water. However, initial PP and PN levels were negatively correlated with TN and TP, indicating an inhibitory effect. Further analysis indicates that PN and PP released from the soil supported the formation of microbial aggregates, enhancing denitrification and phosphorus removal and thus improving water purification over time.

## 1 Introduction

As water levels cyclically change, the Water-Level Fluctuation Zone (WLFZ) within the reservoir experiences alternating wet and dry conditions, which serves as a vital link between terrestrial and aquatic ecosystems and plays a crucial role in nutrient cycling (Chen et al., 2019; He et al., 2017; Sun et al., 2021). The N and P levels in the WLFZ’s overlying water are influenced by various factors. On the one hand, the non-WLFZ soil can significantly influence the N and P levels in the overlying water. During rainfall, runoff can carry biologically available nutrients such as particulate and dissolved N and P in the soil surrounding the non-WLFZ area (Chen et al., 2013; Villa et al., 2014). Consequently, these nutrients are transported into the overlying water of the WLFZ, leading to elevated N and P levels. On the other hand, WLFZ soil also plays a significant role in this regard. Firstly, due to the long-term periodic flooding-drying process, particulate N and P in the WLFZ soil can be directly released into the water due to factors such as hydraulic disturbance (Ran et al., 2020; Wang et al., 2022a, Wang et al., 2022b; Zhang et al., 2022b). Secondly, during flooding, soluble substances such as ammonium nitrogen (Noe and Hupp, 2007) and phosphate ions (Yu et al., 2021) in the soil can quickly dissolve into the overlying water. Additionally, the anaerobic environment formed in the later stages of flooding will further promote the cycling of N and P between soil and water, thereby increasing the flux of N and P released from soil to water (Sun et al., 2021; Yu et al., 2020). These losses of nutrients in the WLFZ soil greatly affects the overlying water’s N and P levels.

The plants in the WLFZ release nutrients such as N and P during flooding, influencing the nutritional status of the overlying water (Chen et al., 2019; Wang et al., 2023). This fundamental process of material cycling in WLFZ has long been the focus of scholarly attention. The decomposition of flooded plants is a complex process influenced by physical, chemical, and biological factors, and the decomposition rate is closely tied to plants’ chemical quality and the surrounding environmental conditions (Lin et al., 2020). In terms of chemical quality, the stoichiometry of nutrient elements and the content of specific substances in plants significantly affect their decomposability. For example, plants with low C: N and C: P ratios often facilitate microbial activity due to the relatively higher N and P content, thereby promoting the decomposition of the plants (Luo et al., 2019). Conversely, Plants with higher levels of recalcitrant substances (such as lignin) can inhibit decomposition by impeding microbial enzyme degradation (Silva et al., 2021). Additionally, Elements such as calcium (Ca) and manganese (Mn) may influence the degradation of lignin by affecting the microbial community, thereby lowering the plants’ decomposition rate (Yue et al., 2021). However, more and more studies have found that aquatic environmental factors during plant flooding also significantly impact their decomposition. For instance, higher water flow rates during flooding can accelerate the decomposition by enhancing the physical breakdown of the plants (Zhai et al., 2021); within specific ranges, elevated temperatures (Tang et al., 2023), higher salinity (Zhai et al., 2020), and increased dissolved oxygen levels (Liu et al., 2022) can also expedite plant decomposition. Additionally, the N and P levels in water may also affect plant decomposition during flooding. Studies conducted by Zhang et al. (Zhang et al., 2022a) and Tie et al (Tie et al., 2022)suggest that in non-flooded conditions, adding N or P fertilizer to soil can enhance microbial activity, thereby accelerating plant decomposition. Consequently, it is reasonable to hypothesize that similar effects may occur under flooded conditions, where N and P levels in water could influence plant decomposition.

During flooding in the WLFZ, particulate nitrogen (PN) and particulate phosphorus (PP) are often released in significant quantities into the overlying water due to hydraulic disturbances and related processes. These particulate forms of N and P not only act as critical nutrient sources but also provide attachment surfaces that facilitate the formation of microbial aggregates, thereby enhancing microbial activity. Although previous studies have explored the roles of PN and PP as nutrient sources and substrates in aquatic environments, their specific effects on plant decomposition and the resulting dynamics of N and P in overlying water under flooding conditions remain poorly understood. Accordingly, we hypothesize that N and P released from soil during the initial stages of flooding—particularly PN and PP—regulate microbial activity, thereby influencing plant decomposition processes and driving dynamic changes in the N and P levels of overlying water.

*Cynodon dactylon* (L.) Pers. was selected as the model plant for this study to test this hypothesis. This perennial herbaceous species is a typical dominant plant in the WLFZ, widely distributed across the Three Gorges Reservoir region and other similar environments. It is characterized by high biomass production and strong adaptability to environmental fluctuations. Moreover, its rapid decomposition makes it a representative species for investigating the effects of plant decomposition on N and P dynamics in overlying water. This study conducted indoor microcosm experiments to simulate 8 different water conditions (experimental waters) based on three experimental variables (water source, sterilization, and soil leaching). Among them, river water represents the natural water environment of the WLFZ, while tap water represents the water commonly used in indoor plant flooding experiments (Hua et al., 2022; Montez et al., 2021; Wu et al., 2023). This comparison helps explore WLFZ soil’s influence on the N and P levels in the overlying water under real-world and simulated conditions. The two original waters mentioned above were employed for soil leaching, stimulating the initial changes in N and P levels in the overlying water during flooding. The sterilization variable was used to examine the influence of water microorganisms. A typical perennial herbaceous plant from the WLFZ, *Cynodon dactylon*, was selected and flooded in the experimental waters mentioned above for flooding experiments. The objective was to investigate the influence of initial N and P levels in the water on the decomposition of *Cynodon dactylon* and the dynamics of N and P levels in the water during flooding.

## 2 Materials and methods

### 2.1 Research area

The Three Gorges Reservoir (TGR) is the largest riverine reservoir in the world (Gao et al., 2016). Since its impoundment in 2010, it has been operated with an annual water level fluctuating between 145 and 175 meters according to the scheduling regulations, forming a WLFZ of approximately 349 km² (Peng et al., 2014). The Pengxi River (30°50′–31°42′N, 107°56′–108°54′E) is one of the main tributaries on the north shore of the TGR, located in the middle reach of the Yangtze River and about 250 km upstream from the TGR (Shi et al., 2017). The basin is under the climate conditions of the North Asian tropical humid monsoon, with an annual average temperature of 18.2 °C and an annual average precipitation of 1053.15 mm (Yue et al., 2016). Due to the fluctuation of water levels in the TGR, the Pengxi River has developed a WLFZ spanning approximately 55.47 km², accounting for 15.9% of the total WLFZ area of the TGR, which is the largest among all tributaries of the TGR (Liu et al., 2023). The flooding period extends from December to June of the following year, totaling approximately 6 months.

### 2.2 Experimental materials

The bermudagrass (*Cynodon dactylon*), the dominant species in the WLFZ in the TGR, was collected and transported to the laboratory. The plant samples were cleaned with ultrapure water and dried in an oven at 105°C for 30 minutes and then at 65°C until reaching a constant weight. The physicochemical properties of plant samples are detailed in **Supplementary Table S1**. The soil samples were collected from the surface (0–30 cm) of the WLFZ in Pengxi River and transported to the laboratory after screening out stones and large plant debris. The physicochemical properties of soil samples are detailed in **Supplementary Table S2**. The water samples of the Pengxi River were collected and transported to the laboratory at 4°C.

### 2.3 Decomposition experiment

First, based on two variables, water source and sterilization status, we obtained 4 types of experimental water: river water (R), tap water (T), sterilized river water (RS), and sterilized tap water (TS). Mix these 4 types of experimental water with soil thoroughly and let it settle for 24 hours. Then, the supernatant was collected to obtain another 4 types of experimental water: soil-leached river water (R(L)), soil-leached tap water (T(L)), soil-leached sterilized river water (RS(L)), and soil-leached sterilized tap water (TS(L)). In total, 8 types of experimental water were obtained.

800ml of each experimental water type was poured into sterilized wide-mouth conical flasks, with a *Cynodon dactylon* decomposition bag placed inside and the water level marked. Each type of experimental water had 4 replicates, and plant samples were collected 5 times throughout the experiment, resulting in a total of 20 sample flasks per type. The entire experiment was conducted indoors under a controlled temperature of 25°C. Corresponding waters were added regularly to maintain a consistent water level, matching the evaporation rate.

Plant samples were collected during the decomposition on days 4, 5, 15, 30, and 60 to determine dry mass, nitrogen, and phosphorus contents. Meanwhile, water samples were collected to determine the contents and forms of N and P. The 60-day experimental duration was selected based on the decomposition dynamics of plant materials. Existing studies indicate that most N and P release from plants occurs during the early to middle stages of decomposition, typically within the first two months. This period is critical for understanding how the nutrient release impacts water quality.

### 2.4 Physiochemical Properties

The dry mass of plant samples was determined by drying them until reaching a constant weight at 65°C. The concentrations of total nitrogen (TN) in plant samples were analyzed using an elemental analyzer (Zuo et al., 2023), while the concentrations of total phosphorus (TP) were determined using the molybdenum blue method after digestion with hydrogen peroxide and concentrated sulfuric acid (Bennett et al., 2003).

The concentrations of total nitrogen (TN) in water samples were determined by UV spectrophotometry after being digested by alkaline potassium persulfate. The concentrations of total dissolved nitrogen (TDN) were determined by the same method using water samples passed a 0.22 μm syringe filter. The concentrations of particulate nitrogen (PN) were calculated based on the difference between TN and TDN. The nitrate nitrogen (NO_3_^-^) concentrations were measured by the spectrophotometric method. The concentrations of ammonium nitrogen (NH_4_^+^) were measured by the salicylic acid-hypochlorite spectrophotometric method (Bureau, 2002).

The concentrations of TP and total dissolved phosphorus (TDP) in water samples were measured by the ammonium molybdate spectrophotometric method. The concentrations of particulate phosphorus (PP) were calculated based on the difference between TP and TDP (Bureau, 2002).

### 2.5 Data and statistical analysis

The residual rates of dry mass (Dt) and nutrient elements (Et) for plant samples were calculated as follows:


(1) $$D_{t}=\frac{M_{t}}{M_{0}}\times 100\%$$

(2) $$E_{t}=\frac{C_{t}\times M_{t}}{C_{0}\times M_{0}}\times 100\%$$

Where M_0_ is the initial dry mass of the plant (g), M_t_ is the remaining dry mass of the plant after t days of flooding (g), C_0_ is the initial concentration of nutrient elements in the plant (mg/g), and C_t_ is the concentration of nutrient elements in the plant on day t (mg/g).

The negative exponential decay model was used to fit the dry mass residual rate of plants (Olson, 1963), and the decomposition rate (k) and the half-life (T1/2) of plant decomposition were estimated as follows:


(3) $$D_{t}=ae^{-kt}$$

(4) $$T_{1/2}=\frac{\ln0.5}{-k}$$

Where: D_t_ is defined in Equation 1; a is the fitting parameter; k is the decomposition rate constant, where a higher value indicates faster decomposition, and vice versa; t is the decomposition time (days).

Significance and correlation analyses were conducted using One-way ANOVA and the Pearson method, respectively, in SPSS (Version 19.0). The fitting of the negative exponential decay model was performed in Microsoft Excel 2019. Redundancy analysis (RDA) was conducted in Canoco (Version 5.0), while Partial Least Squares Structural Equation Modeling (PLS-SEM) analysis was performed in SmartPLS (Version 3.2.9). Graphs were generated in GraphPad Prism (Version 9.5.1).

## 3 Results

### 3.1 Initial N and P concentrations in experimental waters

**Figure 1:**
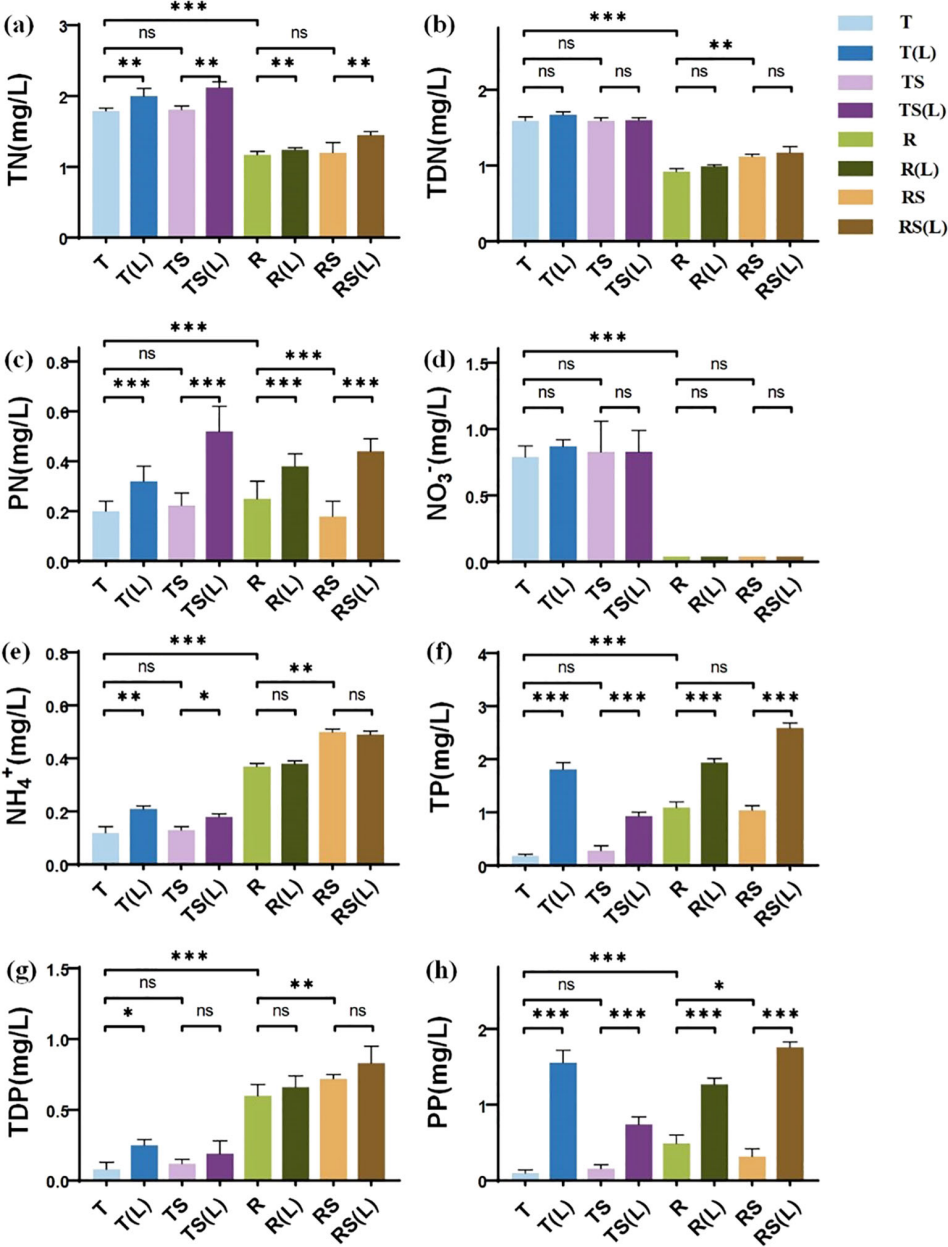
The N and P nutrient concentrations in 8 experimental waters. **(A)** Total nitrogen (TN); **(B)** Total dissolved nitrogen (TDN); **(C)** Particulate nitrogen (PN); **(D)** Nitrate nitrogen (NO₃⁻); **(E)** Ammonium nitrogen (NH₄⁺); **(F)** Total phosphorus (TP); **(G)** Total dissolved phosphorus (TDP); **(H)** Particulate phosphorus (PP). “∗” indicates p< 0.05, “∗∗” indicates p< 0.01, and “∗∗∗” indicates p< 0.001, representing different levels of significant differences in nutrient concentrations between the two types of water. “ns” indicates no significant difference (p > 0.05).

The initial N and P concentrations of 8 experimental waters are presented in **Figures 1A–H**. Significant differences were found between T and R in all forms of N and P. The concentrations of TN, TDN, PN, NO_3_^-^, NH_4_^+^, TP, TDP, and PP in T were 1.79, 1.59, 0.20, 0.79, 0.12, 0.18, 0.08, and 0.10 mg/L, respectively. Those in R were 1.17, 0.92, 0.25, 0.04, 0.37, 1.09, 0.60, and 0.49 mg/L, respectively. River water exhibited significantly lower concentrations of TN, TDN, and NO_3_^-^ (p<0.001), while showing higher concentrations of PN, NH_4_^+^, TP, TDP, and PP (p<0.001) compared to tap water.

Sterilization significantly impacted N and P concentrations of river water, but no significant changes existed in tap water. The concentrations of TDN, PN, NH_4_^+^, TDP, and PP in RS were 1.12, 0.18, 0.04, 0.72, and 0.32 mg/L, respectively. Sterilization significantly reduced the concentrations of PN (p<0.05) and PP (p<0.001), while increasing the concentrations of TDN, NH_4_^+^, and TDP (p<0.01) in river water.

Soil leaching significantly increased the concentrations of PN and PP. The concentrations of PN increased from 0.04, 0.22, 0.25, and 0.18 mg/L in T, TS, R, and RS, respectively, to 0.32, 0.52, 0.38, and 0.44 mg/L in T(L), TS(L), R(L), and RS(L) (p<0.001). Similarly, the concentrations of PP increased from 0.10, 0.16, 0.49, and 0.32 mg/L in T, TS, R, and RS, respectively, to 1.56, 0.74, 1.27, and 1.76 mg/L in T(L), TS(L), R(L), and RS(L) (p<0.001). This suggests that during flooding in the WLFZ, the release of N and P from the soil will significantly impact the N and P content in the overlying water.

### 3.2 The dynamic of plant decomposition

**Figure 2:**
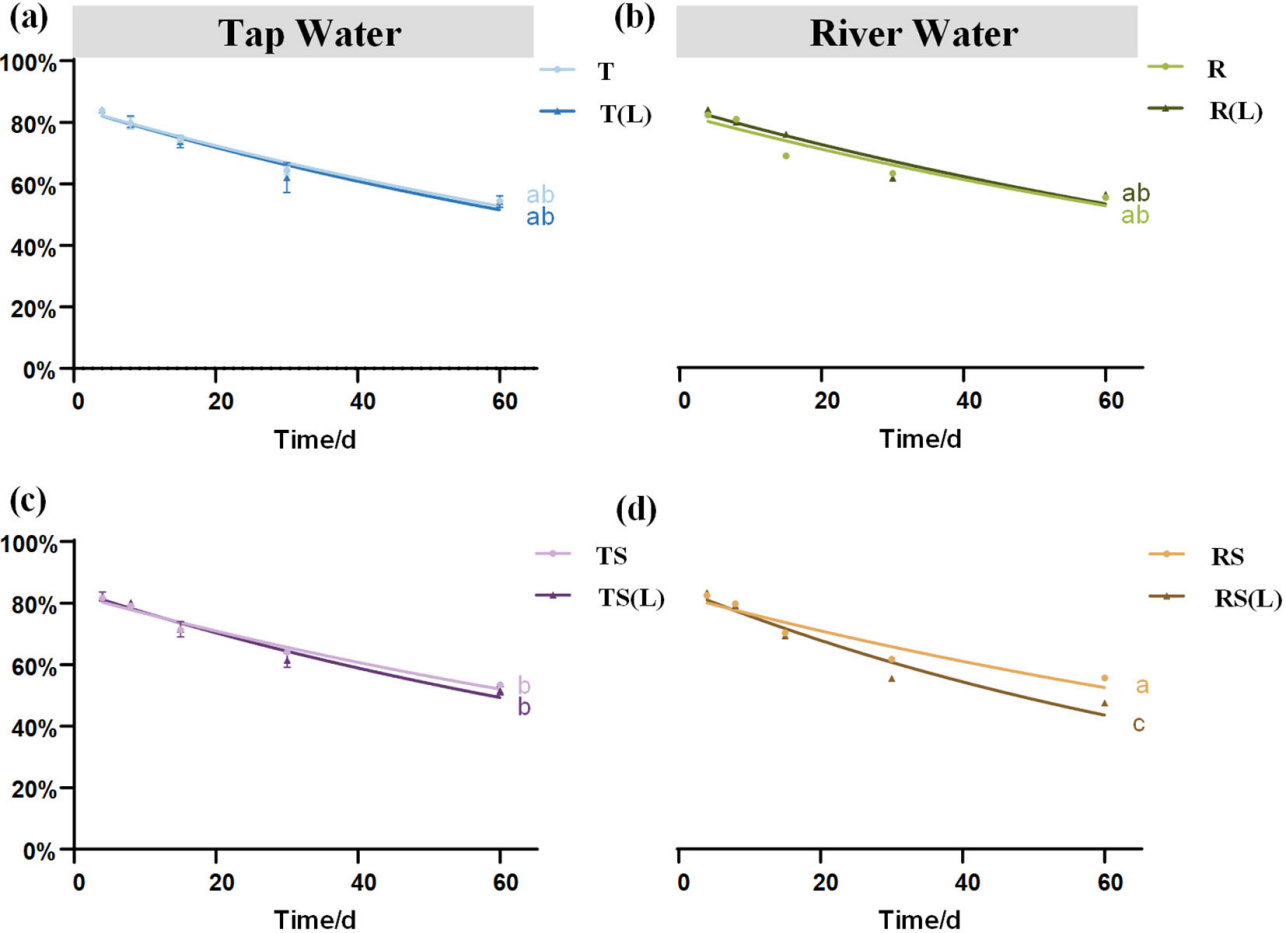
The dynamic changes in the dry mass residual rate of *Cynodon dactylon* in 8 waters. **(A)** Tap water (T) and soil-leached tap water (T(L)); **(B)** River water (R) and soil-leached river water (R(L)); **(C)** Sterilized tap water (TS) and soil-leached sterilized tap water (TS(L)); **(D)** Sterilized-river water (RS) and soil-leached sterilized river water (RS(L)). Different lowercase letters indicate significant differences (P< 0.05) in the dry mass residual rate of *Cynodon dactylon* on Day 60 among 8 waters.

The characteristics of plant dry mass residual rate are presented in **Figures 2A–D**. The dry mass residual rates on day 60 ranks as follows: RS(L) (47.61%)< TS(L) (51.66%)< TS (53.63%)< T(L) (54.22%)< T (54.68%)< R (55.39%)< RS (55.42%)< R(L) (56.29%). A significant difference was found between RS and RS(L) (p<0.05), indicating that after soil leaching, sterilized river water can significantly promote the loss of plant dry mass.

**Figure 3:**
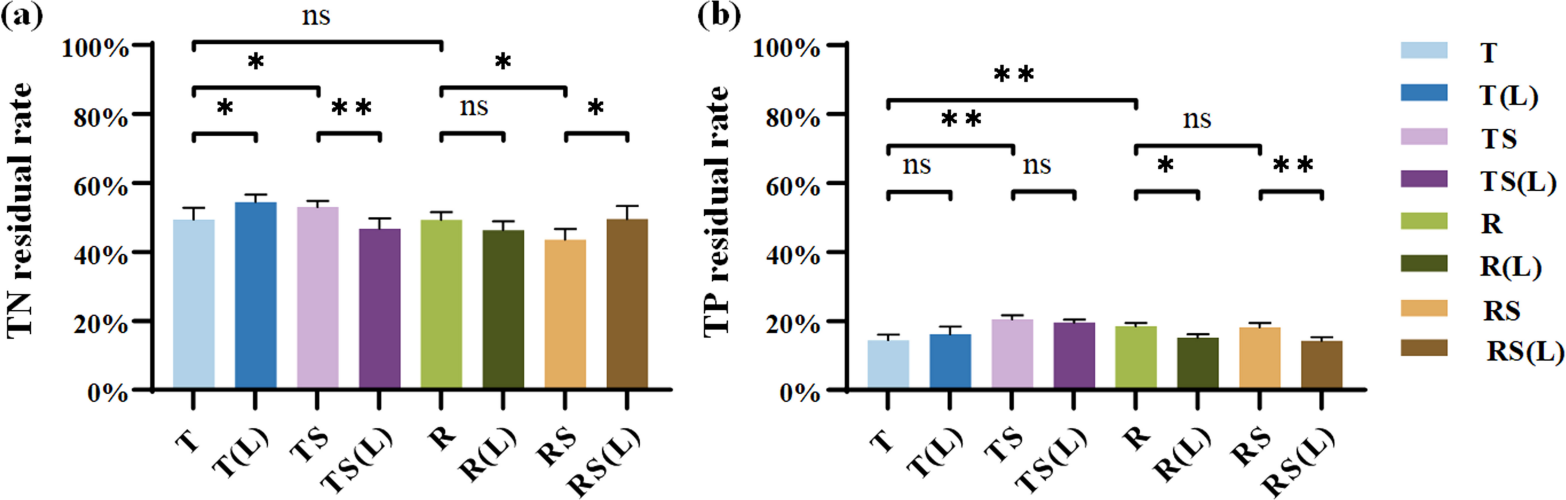
The nutrient residual rates of *Cynodon dactylon* inundated in 8 waters on Day 60. **(A)** Residual rate of total nitrogen (TN); **(B)** Residual rate of total phosphorus (TP). “∗” indicates p< 0.05, and “∗∗” indicates p< 0.01, representing different levels of significant differences in nutrient residual rates of *Cynodon dactylon* between the two types of water, while “ns” indicates no significance (p > 0.05).

The residual rates of TN and TP in the plant on day 60 are presented in **Figures 3A, B**. The TN residual rates followed the order: RS (43.58%)< R(L) (46.39%)< TS(L) (46.82%)< R (49.29%)< T (49.50%)< RS(L) (49.55%)< TS (53.72%)< T(L) (54.48%). The TP residual rates followed the order: RS(L) (14.28%)< T (14.42%)< R(L) (15.18%)< T(L) (16.21%)< RS (18.19%)< R (18.70%)< TS(L) (19.56%)< TS (20.50%).

After soil leaching, waters significantly affect the residual rates of TN and TP in the plant. Regarding TN residual rates, soil leaching increased significantly from 49.50% in T and 43.58% in RS to 54.48% in T(L) and 49.55% in RS(L) (p<0.05). Regarding TP residual rates, leaching reduced significantly from 18.70% in R and 18.19% in RS to 15.18% in R(L) (p<0.05) and 14.28% in RS(L) (p<0.01). This indicates that the changes in N and P levels of experimental water significantly slowed down the release of N while accelerating the release of P from the plant during 60 days of flooding.

The decomposition characteristics of the plant over 60 days in 8 experimental waters are shown in **Table 1**. Soil leaching significantly reduced the half-life of the plant from 90.02 days in TS to 77.88 days in TS(L) (p< 0.05) and from 92.42 days in RS to 63.01 days in RS(L) (p< 0.05). This indicates that the initial N and P content of the experimental water significantly affects the decomposition process of the flooded plant.

**Table 1:** Decomposition characteristics of *Cynodon dactylon* flooded in 8 waters.

Treatment	Decomposition model	k	R^2^	T_1/2_
T	$y=0.8485\times e^{-0.0079t}$	0.0079^bc^	0.9771	87.74^ab^
T(L)	$y=0.8481\times e^{-0.0083t}$	0.0083^bc^	0.9518	83.51^ab^
TS	$y=0.8285\times e^{-0.0077t}$	0.0077^c^	0.9736	90.02^a^
TS(L)	$y=0.8405\times e^{-0.0089t}$	0.0089^b^	0.9665	77.88^b^
R	$y=0.8245\times e^{-0.0075t}$	0.0075^c^	0.9009	92.42^a^
R(L)	$y=0.8460\times e^{-0.0077t}$	0.0077^c^	0.9246	90.02^a^
RS	$y=0.8245\times e^{-0.0075t}$	0.0075^c^	0.9094	92.42^a^
RS(L)	$y=0.8465\times e^{-0.0110t}$	0.0110^a^	0.9382	63.01^c^

Different lowercase letters in the same column indicate significant differences between the indicators (p<0.05).

### 3.3 Dynamics of N and P concentrations in overlying waters

**Figure 4:**
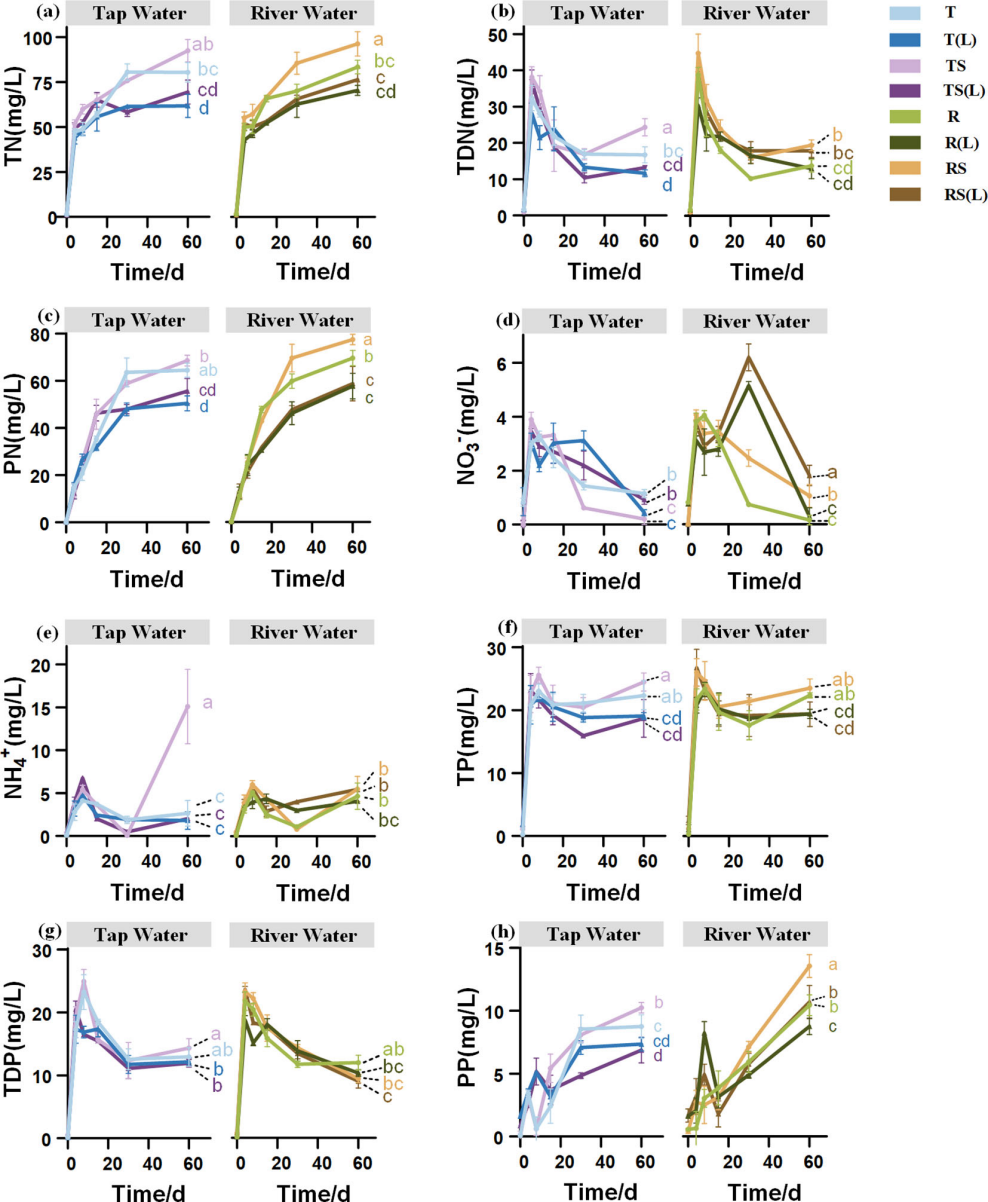
The dynamic changes of N and P concentrations in 8 overlying waters. **(A)** Total nitrogen (TN); **(B)** Total dissolved nitrogen (TDN); **(C)** Particulate nitrogen (PN); **(D)** Nitrate nitrogen (NO₃⁻); **(E)** Ammonium nitrogen (NH₄⁺); **(F)** Total phosphorus (TP); **(G)** Total dissolved phosphorus (TDP); **(H)** Particulate phosphorus (PP). Different lowercase letters indicate significant differences (P< 0.05) in the nutrient concentrations on Day 60 among 8 overlying waters.

The dynamics of N and P concentrations appear to be similar across various waters (**Figures 4A–H**). Regarding N, TN and PN concentrations increased throughout the experiment, and the growth slowed after day 30. The concentrations of TDN rapidly increased during the early stage and decreased from day 4. From day 30 to day 60, opposite trends were observed in the NO_3_^-^ and NH_4_^+^ concentrations, with the NO_3_^-^ concentrations decreasing while NH_4_^+^ concentrations were increasing. Regarding P, the TP, TDP, and PP concentrations experienced rapid initial increases, peaking between 4 and 8 days, followed by a slight decrease. However, the later trends differ: TDP concentration continues to decline slowly, whereas PP and TP concentrations continue to rise.

The source of N and P in experimental water significantly affects the concentrations of N and P in the overlying water on day 60. In group R, the concentrations of NH_4_^+^, NO_3_^-^, and PP were 4.67, 0.15, and 10.43 mg/L, respectively. Those in group T were 2.64, 1.17, and 8.67 mg/L, respectively. Group R exhibited higher levels of NH_4_^+^ and PP (p<0.05) and lower levels of NO_3_^-^ (p<0.05) than group T.

The sterilization of experimental water significantly affects the concentrations of N in the overlying water on day 60. In group RS, the concentration of TDN was 19.48 mg/L on day 60 (**Figure 4B**), significantly higher than that in group R (13.79 mg/L) (p<0.05).

The N and P released from the soil into the initial water can significantly influence the concentrations of N and P in the overlying water on day 60. The concentrations of TN in the T, TS, R, and RS are 80.60 mg/L, 91.15 mg/L, 83.22 mg/L, and 96.55 mg/L, respectively, significantly (p< 0.05) higher than those in T(L), TS(L), R(L), and RS(L), which are 62.30 mg/L, 69.00 mg/L, 70.34 mg/L, and 76.54 mg/L (p<0.05), respectively. The concentrations of TP in TS, R, and RS are 24.55 mg/L, 22.33 mg/L, and 23.08 mg/L, respectively, significantly (p< 0.05) higher than those in TS(L), R(L) and RS(L), which are 18.73 mg/L, 19.09 mg/L, and 19.68 mg/L (p<0.05), respectively. This indicates that when a portion of N and P in the initial overlying water originates from soil release, the concentrations of TN and TP in the overlying water will significantly decrease after 60 days of plant flooding.

## 4 Discussion

### 4.1 The impact of water source on N and P levels in experimental water

The forms of N and P exhibit significant differences between natural water and tap water. In the context of three experimental variables, the water source explained 42.3% of the total variation in N and P levels across all water samples (**Supplementary Table S3**). Notably, tap water exhibited significantly lower PN, PP, and TDP levels than natural water (**Figures 1C, G, H**). This is because tap water undergoes processes such as coagulation, sedimentation, and filtration during the treatment process, significantly reducing the PN and PP in the water (Zaki et al., 2023). Moreover, adding coagulants rich in metal cations during coagulation prompts a reaction with phosphates in the water (Zong et al., 2022). This reaction leads to the formation of insoluble precipitates, subsequently eliminating phosphates from the water, thereby significantly reducing TDP levels. Previous studies often used tap water instead of natural water to simulate plant decomposition experiments (Hua et al., 2022; Liu et al., 2017; Montez et al., 2021; Wu et al., 2023). However, our research discovered that tap water has significantly lower TP and PP content; thus, replacing natural water with tap water in plant flooding experiments does not reflect the actual nutrient environment. This point has been affirmed by Ping (Ping et al., 2017) and Pan et al (Pan et al., 2017), who found significant differences in plant decomposition and N and P release when plants were flooded in natural water compared to tap water. One reason is that decomposers’ nutrient demands often exceed plants’ nutrient supply (Rawlik et al., 2021). In such cases, higher N and P inputs in the environment can promote microbial biomass and enhance its activity, thus accelerating the N and P cycling of plants (Zhang et al., 2019). Therefore, using tap water instead of natural water in plant flooding decomposition experiments may underestimate the release of N and P from plants in natural environments.

**Figure 5:**
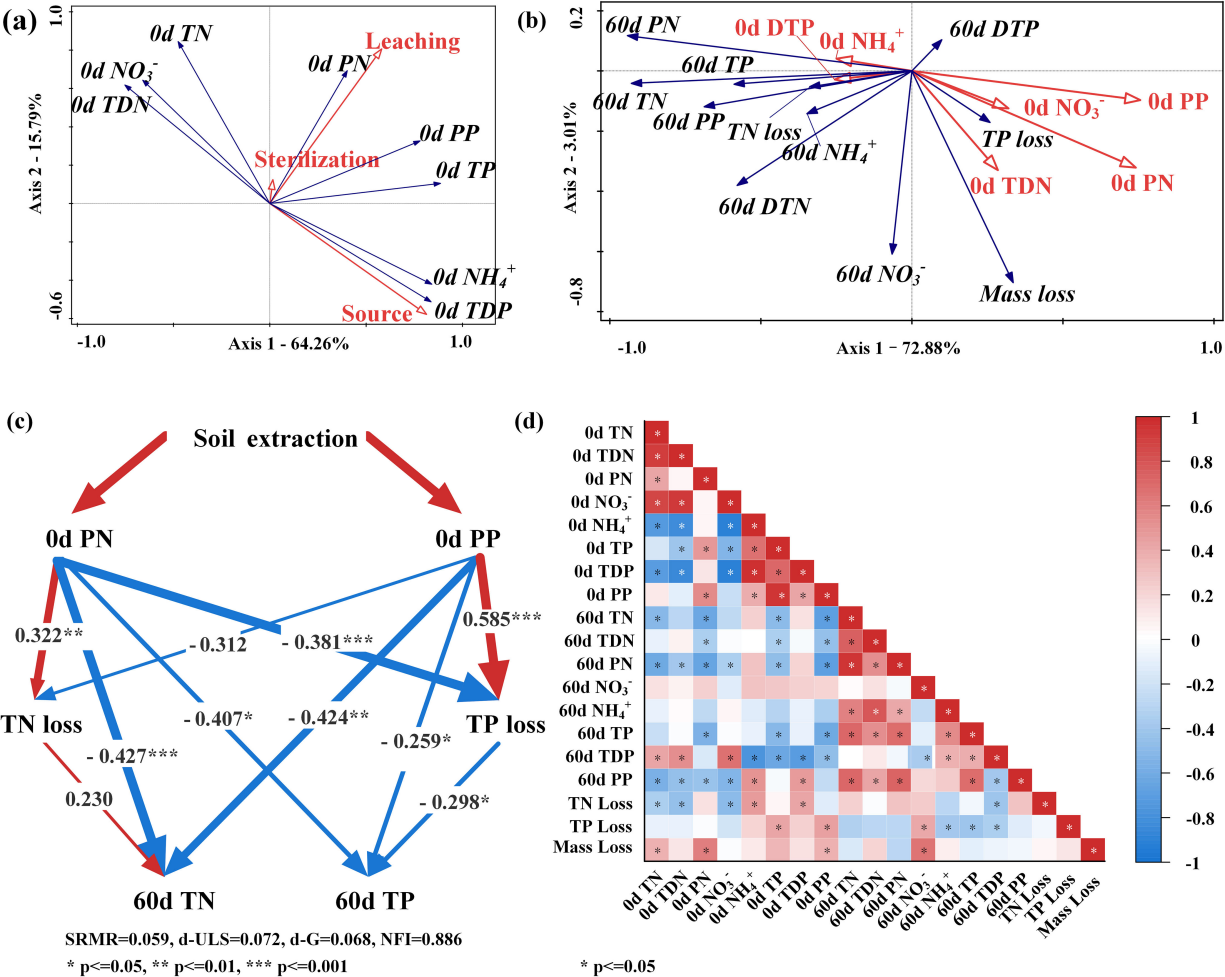
Analysis of the relationship between the initial N and P concentrations in the experimental waters and the decomposition status of the flooded plant. **(A)** RDA analysis: The effects of three experimental variables on N and P concentrations in experimental waters. **(B)** RDA analysis: The effects of initial water’ N and P concentrations on 60-day plant dry mass loss, plant N loss, plant P loss, and overlying waters’ N and P concentrations. **(C)** PLS-SEM analysis: The effect of soil leaching on the TN and TP concentrations in 60-day overlying waters. The red arrows indicate the positive effect, the blue arrows indicate the negative effect, and the numbers on the arrows indicate the explanatory rates. **(D)** Correlation analysis of initial N and P nutrient concentrations in experimental waters, N and P nutrient concentrations in 60-day overlying waters, 60-day plant dry mass loss, 60-day plant N loss, and 60-day plant P loss.

The release of soil nutrients significantly affects the concentration of N and P in the overlying water of the WLFZ, mainly increasing the particulate N and P content in the water. In this experiment, a water-to-soil ratio as high as 7.5:1 was used for soil leaching, simulating the release of soil N and P when the overlying water fully interacts with the soil during flooding. The results show that although soil leaching has little effect on the content of soluble substances TDN and TDP, it significantly increases the content of particulate substances PN and PP in the water (**Figures 1C, H**). The RDA analysis also indicates that soil leaching primarily increases the content of PN and PP in the water (**Figure 5A**). Cheng et al. (Cheng et al., 2021) found that complete interaction between water and soil results in the loss of soil N and P, primarily in particulate form. Additionally, the larger the volume of water, the higher the proportion of PN and PP (Wang et al., 2025). This is due to the hydraulic disturbance, which can quickly resuspend particles into the water. Similar phenomena also frequently occur during flooding in the WLFZ, exacerbating the release of soil PN and PP (Nsabimana et al., 2021; Zhang et al., 2021c). Therefore, when simulating the plant flooding decomposition in the WLFZ, it is crucial to consider the initial concentrations of PN and PP in the overlying water to replicate natural conditions faithfully.

### 4.2 Characteristics of *Cynodon dactylon* decomposition across experimental waters

During flooding, the WLFZ soil introduces various forms of N and P, such as TP, PP, TDP, and PN, into the overlying water, thereby promoting the decomposition of *Cynodon dactylon* and the release of P from it. In this experiment, the decomposition rate of *Cynodon dactylon* significantly accelerates when submerged in soil-leached water. The RDA analysis indicates that the loss of *Cynodon dactylon* dry mass is mainly promoted by TDN and PN in the experimental water, and the loss of *Cynodon dactylon* TP is promoted by PN and PP (**Figure 5B**). Similar phenomena have been observed by Du et al (Du et al., 2014), suggesting that higher levels of N and P in water tend to facilitate plant flooding decomposition and P release. Firstly, the TN and TP concentrations of the experimental waters ranged from 1.10 mg/L to 1.95 mg/L and from 0.23 mg/L to 2.50 mg/L, respectively, indicating a relative scarcity of N and P nutrients in the water, which may limit microbial growth. Therefore, waters with higher levels of N and P forms, such as TDN, PP, and PN, can provide richer nutrients for microorganisms, promoting their growth and reproduction (Zhang et al., 2021a). With more microorganisms consuming TDN, PP, and PN in the water, the balance of N and P in the water is disrupted, thus accelerating the decomposition and the release of N and P from *Cynodon dactylon* into the water. Secondly, higher initial P content can also increase phosphatase activity (Margalef et al., 2017), promoting the mineralization of organic phosphorus (Li et al., 2021), directly resulting in faster decomposition of *Cynodon dactylon* and release of P.

During flooding, the soil in the WLFZ releases additional N compounds, such as TN and PN, into the overlying water. This exacerbates N enrichment within *Cynodon dactylon* during decomposition, resulting in a slower rate of N release. We discovered that the decomposition rate of *Cynodon dactylon* significantly accelerates when flooded in soil-leached sterilized river water; however, at the same time, the release of its TN content was notably slower (**Figure 3A**). This phenomenon is related to the levels of PN and TDN in the experimental water. RDA analysis indicates that while PN and TDN promote the loss of *Cynodon dactylon* dry matter, they simultaneously inhibit TN release (**Figure 5B**). Pichon et al. (Pichon et al., 2020) also found that although N content in decomposition environments can significantly accelerate plant dry mass loss, it can also promote N accumulation in plants, which is consistent with our study. This seemingly contradictory phenomenon is primarily caused by the enrichment of N by microorganisms attached to plant tissues. It is generally believed that the plant’s C: N ratio strongly influences the decomposition rate of plants. A higher C: N ratio indicates relative N deficiency in the plant tissues, which is unfavorable for the growth and reproduction of microorganisms attached to plants (Xie et al., 2022). In such situations, microorganisms tend to enrich N from the environment to alleviate nitrogen limitation. In this experiment, the C: N ratio of *Cynodon dactylon* was 35.14 (**Supplementary Table S1**), significantly surpassing the commonly accepted threshold of 25 (Pei et al., 2019). This indicates a N deficiency in *Cynodon dactylon*, thereby inhibiting microbial growth. However, we found that the soil-leached experimental waters contained higher levels of N. On the one hand, the increased N content promotes the growth and reproduction of microorganisms attached to plants, thereby accelerating the decomposition of carbohydrates, proteins, et al., existing less residual matter. On the other hand, these N elements are not only enriched in the microorganisms attached to the plant body but also fixed in the microbial byproducts remaining within the plant body (Harindintwali et al., 2020), hence resulting in a slower N release rate of *Cynodon dactylon*.

### 4.3 Dynamic characteristics of N and P concentrations across overlying waters

The initial nutrient differences in the overlying waters do not affect the changing patterns of N and P concentrations in the overlying water. This experiment revealed similar concentration dynamics in all forms of N and P across different waters. In each kind of water, TDN and TDP concentrations both peaked at 4 days (**Figures 4B, G**), indicating the rapid release of soluble substances from *Cynodon dactylon*. However, there was a significant decrease in TDN and TDP concentrations between 4 and 30 days, which may be attributed to microbial absorption and transformation of nutrients in the water (Kurniawan et al., 2021). The Concentrations of TN, TDN, PN, TP, and TDP gradually stabilized after the 30^th^ day (**Figures 4A–C, F, G**), and the decomposition of plants and microbial utilization in the system eventually reached a dynamic equilibrium over time. The changes in NH_4_^+^ and NO_3_^-^ concentrations were more complex. In the early stage, the concentrations of NH_4_^+^ and NO_3_^-^ fluctuated significantly, which is because NH_4_^+^ is highly unstable and readily oxidizes into NO_3_^-^ or converts into volatile-free ammonia (Zhang et al., 2021b). After the 30th day, it was observed that the concentration of NH_4_^+^ in the overlying water gradually increased, while the concentration of NO_3_^-^ decreased gradually (**Figures 4D, E**). This is attributed to microbial oxygen consumption, gradually reducing dissolved oxygen concentration in the water, creating a hypoxic environment (Zhang et al., 2017). On one hand, the hypoxic conditions inhibit the nitrification process, leading to a decrease in NH_4_^+^ consumption. Consequently, the rate of NH_4_^+^ release from plants surpasses the rate of NH_4_^+^ consumption by microbes, resulting in an elevation of NH_4_^+^ concentration in the overlying water. On the other hand, hypoxia promotes denitrification, leading to a continuous decrease in NO_3_^-^.

During flooding in the WLFZ, changes in the N and P content of the overlying water, induced by soil nutrient release, may lead to lower N and P concentrations in the overlying water during the later stages of flooding. This could be attributed to the stimulation of water microbial growth by the particulate N and P released from the WLFZ soil, thereby enhancing the water’s self-purification capacity. We observed that at day 60, the concentrations of TN and TP in the soil-leached waters were significantly higher than those in the corresponding non-leached waters (**Figures 4A, F**). However, the TN and TP loss rates in *Cynodon dactylon* did not exhibit the same pattern in leached and non-leached systems (**Figures 3A, B**). Further correlation analysis indicated that the amount of N and P released from the plant was not positively correlated with the N and P concentrations in the overlying water (**Figure 5D**). Yuan et al. (Yuan et al., 2014) found similar decoupling phenomena in the WLFZ, suggesting that during flooding in the WLFZ, the N and P concentrations in the overlying water may be dominated by other factors. We observed that soil leaching significantly raised PN and PP levels in the water (**Figure 5A**), accounting for much of the variation in TN and TP concentrations on the 60th day(**Figure 5B**). Based on these observations, we constructed a structural equation model (**Figure 5C**) to investigate how the initial PN and PP concentrations influence TN and TP concentrations in the overlying water on the 60th day. We found that, among the 4 pathways influencing TN and TP concentrations, the direct effects of the 3 pathways significantly outweighed the indirect effects (**Supplementary Figure S1**). This suggests that the initial PN and PP primarily impact TN and TP levels in the overlying water through direct mechanisms within the water body, rather than indirectly affecting plant release. We speculate this relates to the aggregate observed in the overlying water at day 60. At the end of the experiment, a substance resembling microbial aggregates was found in the overlying water of all systems (**Supplementary Figure S2**), particularly pronounced in the leaching systems. Considering that microbial communities in water are proficient at denitrification and phosphorus removal (Fallahi et al., 2021; Zhao et al., 2022), we speculate that the presence of PN and PP in leaching systems could stimulate the formation of these microbial aggregates, leading to lower N and P levels in the overlying waters.

Numerous related studies support our idea. On the one hand, elevated levels of PN and PP can serve as essential nutrients for microorganisms, particularly in water deficient in N and P. Consequently, this stimulates microbial growth (Wang et al., 2018b) and induces changes in microbial structure (Yang et al., 2022), thereby enhancing their abilities in denitrification and dephosphorization (He et al., 2020). For example, when dealing with wastewater deficient in N and P from paper mills, it’s common practice to introduce suitable amounts of N and P into the water (Cai et al., 2019). This supplementation stimulates the proliferation of microorganisms, consequently enhancing their effectiveness in removing N and P from the water. On the other hand, the particulate nature of PN and PP provides a substrate for attachment and aggregation (Fan et al., 2023), thereby forming efficient reactors composed of particles and microorganisms that play a critical role in the cycling of N and P (Walch et al., 2022). Within these aggregates, redox gradients create favorable conditions for denitrification by facultative anaerobic microorganisms (Chen et al., 2017), while their adhesive polymers adsorb and immobilize phosphorus in the water (He et al., 2018), ultimately contributing to significant reductions in TN and TP concentrations in the overlying water.

However, in the actual environment of the WLFZ, water level fluctuations may regulate the structural stability of aggregates and microbial activity through changes in dissolved oxygen and hydrodynamic disturbances. For example, dynamic changes in dissolved oxygen may alter the redox gradients within aggregates, thereby affecting denitrification efficiency (Wang et al., 2018a). At the same time, water flow disturbances may disrupt the structural integrity of aggregates (Wu et al., 2012) or influence their suspension and sedimentation behavior (Luo et al., 2022). These processes may collectively influence microbial attachment, nutrient transformation efficiency, and the overall purification capacity of the system. Therefore, future studies should integrate dynamic hydrological conditions to systematically evaluate the mechanisms and purification performance of PN and PP under fluctuating water levels.

The N and P levels in water are influenced by dynamic equilibrium processes constituted by both plant release and microbial uptake. If we only consider plant release, the N and P concentrations in the overlying waters should positively correlate with the amount of N and P released by plants on the 60th day. However, our experiment did not observe this correlation, indicating that microbial uptake and other factors may predominate N and P variances in the overlying waters. Thus, the release of PN and PP from the WLFZ soil in the early stages of flooding, along with the subsequent denitrification and phosphorus removal by microbial aggregates, significantly influences the N and P content of the overlying water and should not be underestimated.

## 5 Conclusion

This study investigates the changes in initial N and P levels in the overlying water during flooding in the WLFZ, focusing on its effects on the decomposition of *Cynodon dactylon* and the dynamics of N and P in the overlying water during this process. Results indicate that flooding significantly elevated N and P levels in the initial overlying water, particularly in the forms of particulate nitrogen (PN) and particulate phosphorus (PP). These changes likely impacted plant decomposition and the fluctuations in N and P concentrations in the water. After 60 days of flooding, the residual dry matter, total nitrogen (TN), and total phosphorus (TP) of *Cynodon dactylon* were 47.61%-56.29%, 43.58%-54.48%, and 14.28%-20.50%, respectively. Initial PN and total dissolved nitrogen (TDN) promoted the dry mass loss of *Cynodon dactylon*, while initial PN and PP promoted TP loss; however, TN loss was inhibited by initial PN and TDN. By day 60, no positive correlation was observed between the N and P released from the plant and the TN and TP concentrations in the overlying water. In contrast, initial PP and PN concentrations were negatively correlated with TN and TP concentrations on day 60, suggesting an inhibitory effect. Further analysis revealed that PN and PP from the soil promoted microbial aggregate formation in the overlying water. These aggregates exhibited denitrification and phosphorus removal capacities, enhancing the water’s self-purification ability. Consequently, N and P levels in the water decreased after 60 days of plant flooding.

## Data Availability

The datasets presented in this study can be found in online repositories. The names of the repository/repositories and accession number(s) can be found below: https://doi.org/10.5061/dryad.zcrjdfnpd.
