# Supplementary material for: Interactions between N, P in the overlying water and flooding-induced decomposition of Cynodon dactylon in the water-level fluctuation zone
Source: Front Plant Sci. 2025 Feb 10;16:1526507. doi: 10.3389/fpls.2025.1526507 (PMC11847792; doi:10.3389/fpls.2025.1526507)
Supplement: Supplementary file 1 [file DataSheet1.docx]

Supplementary Material

# Supplementary Figures and Tables

## Supplementary Figures


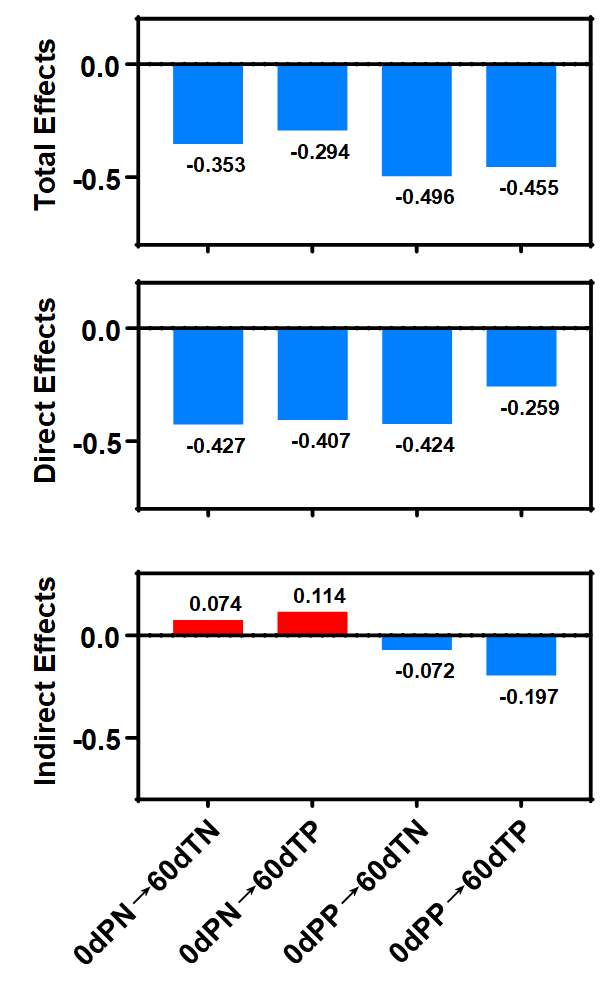


**Supplementary Figure S1.** The total effects, indirect effects, and direct effects of the PLS-SEM analysis.


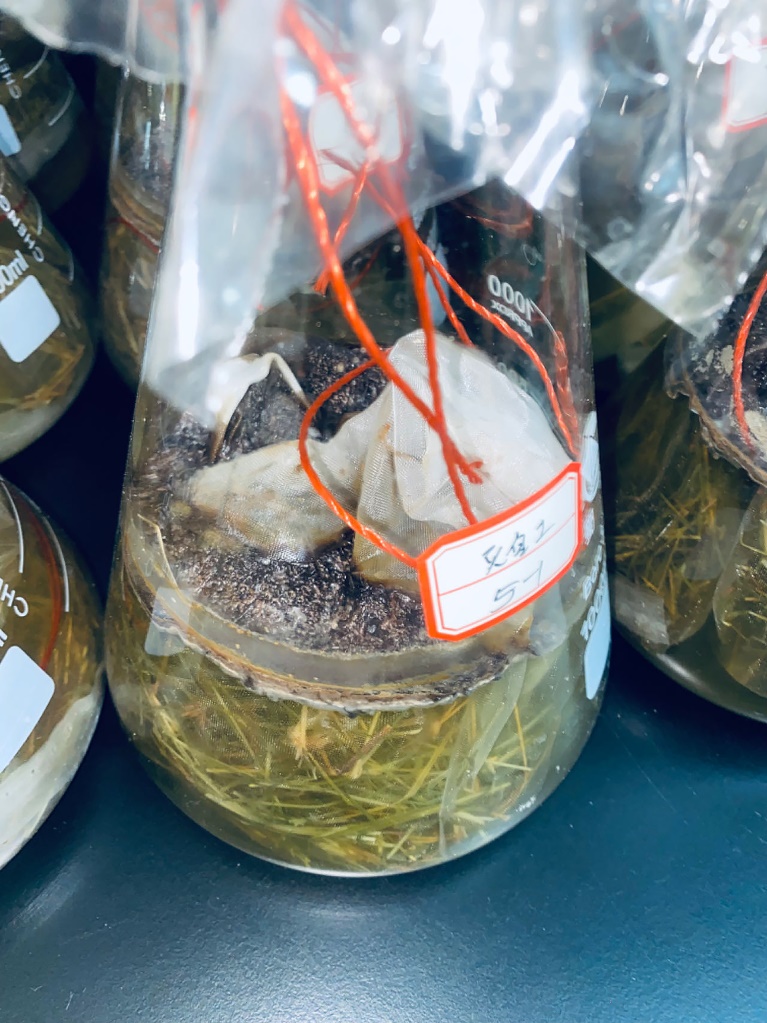


**Supplementary Figure S2.** The state diagram of the leaching system on the 60^th^ day.

## Supplementary Tables

**Supplementary Table S1.** Physicochemical properties of the plant samples

| Water Content（%） | Total Carbon（mg·kg^-1^） | Total Nitrogen（mg·kg^-1^） | Total Phosphorus（mg·kg^-1^） | C/N |
| --- | --- | --- | --- | --- |
| 61.2 ± 1.8 | 466.66 ± 8.58 | 13.28 ± 0.48 | 2.08 ± 0.14 | 35.14± 1.82 |

**Supplementary Table S2.** Physicochemical properties of the soil samples for leaching

| Soil PH | Moisture Content（%） | | TC  （g·kg^-1^） | TN  （g·kg^-1^） | TP  （g·kg^-1^） | AP  （mg·kg^-1^） |
| --- | --- | --- | --- | --- | --- | --- |
| 6.88 ± 0.02 | | 61.2 ± 1.8 | 23.39 ± 0.92 | 1.70 ± 0.03 | 0.68 ± 0.03 | 3.26 ± 0.08 |

**Supplementary Table S3.** The RDA analysis results of the effects of three experimental variables on N and P concentrations in experimental waters.

| Variables | Explains % | Contribution % | Pseudo-F | P |
| --- | --- | --- | --- | --- |
| Source | 42.3 | 47.9 | 24.8 | 0.002 |
| Soil Leaching | 45.2 | 51.2 | 98.5 | 0.002 |
| Sterilization | 0.8 | 0.9 | 1.8 | 0.170 |
